# PROTECTIVE EFFECT OF SHINGLES VACCINE ON ALZHEIMER’S DISEASE CAN BE MODULATED BY RISK VARIANT OF NECTIN2 GENE

**DOI:** 10.1093/geroni/igad104.0413

**Published:** 2023-12-21

**Authors:** Svetlana Ukraintseva, Hongzhe Duan, Rachel Holmes, Deqing Wu, Igor Akushevich, Arseniy Yashkin, Konstantin Arbeev, Anatoliy Yashin

**Affiliations:** Duke University, Durham, North Carolina, United States; Social Science Research Institute, Duke University, Durham, North Carolina, United States; Social Science Research Institute, Duke University, Durham, North Carolina, United States; Social Science Research Institute, Duke University, Durham, North Carolina, United States; Duke University, Durham, North Carolina, United States; Duke University, Durham, North Carolina, United States; Social Science Research Institute, Duke University, Durham, North Carolina, United States; Social Science Research Institute, Duke University, Durham, North Carolina, United States

## Abstract

The aging-related decline in immune surveillance was suggested to play a major role in Alzheimer’s disease (AD). Adult vaccinations may support the aging immune system beyond prevention of the target disease, and through this provide a protection against AD. Such off-target effects of vaccines may potentially be affected by genetic risk factors for AD. To explore this possibility, we estimated association of the vaccination against shingles (reactivated herpes zoster virus infection) received between ages 65-75 with AD onset at ages 75+ in the Health and Retirement Study (HRS) survey participants, comparing the carriers and non-carriers of rs6859 A allele of NECTIN2 gene (AD risk factor that is also involved in vulnerability to viral infections). We used logistic regression model with covariates including sex, race, education, and smoking status. We found that shingles vaccine was significantly associated with lower odds of AD onset later in life, in the total sample (p-value=0.021; OR=0.55 (0.33, 0.92)). After stratification by genotype, the protective effect of vaccine became stronger in rs6859 A carriers (p-value=0.016; OR=0.44 (0.22, 0.86)), but disappeared in the non-carriers, for both significance and size (p-value=0.81; OR=0.90). We conclude that adult shingles vaccine may reduce chances of AD at older ages, and this effect appears more beneficial for carriers of rs6859 A allele of NECTIN2 gene. Repurposing of existing vaccines may benefit the aging immune system, and be a promising approach to personalized genotype-tailored prevention of AD.

